# Spectres of Clock Evolution: Past, Present, and Yet to Come

**DOI:** 10.3389/fphys.2021.815847

**Published:** 2022-02-11

**Authors:** Maria Luísa Jabbur, Carl Hirschie Johnson

**Affiliations:** Department of Biological Sciences, Vanderbilt University, Nashville, TN, United States

**Keywords:** evolution of circadian clocks, circadian rhythms, bacterial rhythms, evolution and climate change, photoperiodism, biological timekeeping

## Abstract

Circadian clocks are phylogenetically widespread biological oscillators that allow organisms to entrain to environmental cycles and use their steady-state phase relationship to anticipate predictable daily phenomena – such as the light-dark transitions of a day – and prepare accordingly. Present from cyanobacteria to mammals, circadian clocks are evolutionarily ancient and are thought to increase the fitness of the organisms that possess them by allowing for better resource usage and/or proper internal temporal order. Here, we review literature with respect to the ecology and evolution of circadian clocks, with a special focus on cyanobacteria as model organisms. We first discuss what can be inferred about future clock evolution in response to climate change, based on data from latitudinal clines and domestication. We then address our current understanding of the role that circadian clocks might be contributing to the adaptive fitness of cyanobacteria at the present time. Lastly, we discuss what is currently known about the oldest known circadian clock, and the early Earth conditions that could have led to its evolution.

## Introduction

This article presents evidence and speculations regarding the evolution of circadian clocks with an organisation based on the famous novella by Charles Dickens, *A Christmas Carol*, where the protagonist is visited by the Spectres of Christmas Past, Present, and Yet to Come. In this review, we will use the terms “ghost” and “spectre” interchangeably and address the timing of the spectral topic of clock evolution in the reverse order from Master Dickens: Evolution Yet to Come, Adaptive Value in the Present, and the Clock Primaeval.

We do not know when the first circadian clock evolved, but it is reasonable to suppose that it might have occurred in blue green-algae, also known as cyanobacteria, because they are the oldest extant species (2,000–3,000 Mya) whose present-day descendants are known to comprise an undisputed circadian system. Moreover, the ecological pressures (esp. light and temperature cycles) that are likely to have selected for and maintained circadian systems have been present on earth since before cyanobacteria originated (albeit the early earth is likely to have spun with a higher frequency than its current 24 h cycle). Their post-translational oscillator, which is composed of the clock proteins KaiA, KaiB, and KaiC, is a remarkably simple self-sustained oscillator system that is capable of producing robust, temperature-compensated, ∼24 h oscillations of KaiC phosphorylation – promoted by KaiA and inhibited by KaiB (Williams et al., 2002; Nakajima et al., 2005; Johnson et al., 2017). In turn, these oscillations – acting through the transcription factor RpaA – lead to an almost global circadian control of gene expression (Liu et al., 1995; Markson et al., 2013). Cyanobacteria have been crucial to our understanding of the inner and outer workings of the circadian clock; due in part to the post-translational nature of their oscillator (Tomita et al., 2005; Rust et al., 2007) and the fact that the interactions of clock proteins amongst themselves and with DNA can be studied *in vitro* (Nakajima et al., 2005; Chavan et al., 2021), we have now atomic-level resolution of clock proteins and their interactions (Pattanayek et al., 2004; Ye et al., 2004; Iwase et al., 2005; Johnson et al., 2008; Abe et al., 2015; Tseng et al., 2017; Hong et al., 2018; Mori et al., 2018), and due to cyanobacteria’s fast life cycle and ease of growth, we can use them to demonstrate that having a circadian clock is advantageous by *direct* assessments of fitness (Ouyang et al., 1998; Woelfle et al., 2004; Ma et al., 2013).

While the circadian clocks of cyanobacteria are currently well-studied and characterised, the field of prokaryotic chronobiology in general is still in its infancy (Johnson et al., 2017); except for cyanobacteria, we do not know much about whether circadian clocks exist in other Eubacteria or in Archaea, and if so, whether they have properties that are different from those in cyanobacteria or in eukaryotes. So far, only two Eubacteria outside of Cyanobacteria have been reported to exhibit circadian-like properties: *Klebsiella aerogenes* (Paulose et al., 2016, 2019) and *Bacillus subtilis* (Eelderink-Chen et al., 2021). And while there have been reports of diurnally synchronised rhythmicity in *Halobacterium*, some of which appear to persist in constant darkness, other features of circadian clocks – such as temperature compensation – have yet to be tested in an Archaeon (Whitehead et al., 2009; Maniscalco et al., 2014). This is not too surprising since the study of circadian clocks in prokaryotes is rather new. For example, the discovery of circadian rhythms in cyanobacteria only occurred in the late 1980s (Grobbelaar et al., 1986; also see Johnson et al., 1996; Huang and Lin, 2009 for historical panoramas), much after plants (1729–1825, Sweeney, 1987), fungi (Pittendrigh et al., 1959), and even mammals (Richter, 1922). Prior to the cyanobacterial studies in the 1980–1990s, two studies had attempted to demonstrate circadian rhythms in prokaryotes, specifically *Escherichia coli* (Halberg and Conner, 1961) and *Klebsiella* (Sturtevant, 1973), but unlike the cyanobacterial work, neither of those papers managed to persuade chronobiologists that the apparent rhythms were *bona fide* circadian phenomenon, largely because they failed to demonstrate the defining properties of circadian rhythms of temperature compensation and entrainment (Johnson et al., 1996).

The long lag from the discovery of circadian phenomena in eukaryotes to that in prokaryotes was largely due to a bias among chronobiologists that reflected the pre-1990, organism-centric idea that organisms with life cycles shorter than a day – such as is true for many bacteria – would have no use for a circadian oscillator, as they would go through multiple generations within a single day (Pittendrigh, 1993; Johnson et al., 1996; Johnson and Rust, 2021). Since no single cell would be able to see the entirety of a day, it seemed reasonable to suppose that there would be no selection for a daily timer. If, however, we think not only at the level of the organism or the cell, but also at a group level (i.e., multiple generations, an entire bacterial lineage), the adaptiveness of both daily and seasonal timekeepers for short-lived organisms becomes apparent: while a single bacterial cell might not see an entire day (let alone an entire year) before cell division, the bacterial lineage will. Using the example of photoautotrophic cyanobacteria that are totally dependent upon sunlight for energy, one can easily imagine that a failure to anticipate nighttime for any cyanobacterial population could be catastrophic for its ability to survive and reproduce, particularly as they need to produce sufficient glycogen reserves if they are to last through the night (see Lambert et al., 2016). The fact that cyanobacteria can transmit circadian phase to their daughter cells unimpeded through multiple cell division cycles likely facilitates this anticipation of key environmental transitions such as sunsets and sunrises (Mihalcescu et al., 2004). Of note, the phenomenon of a species being able to time itself progressively through cycles that last longer than their generational time is not exclusively prokaryotic, and has been well-studied in eukaryotes: monarch butterfly migration relies on seasonal photoperiodic cues and spans up to five generations to complete the migratory cycle (Reppert and de Roode, 2018; Guerra, 2020); similarly, diapause in many insects has been shown to be a transgenerational response (Denlinger, 1998). Cyanobacteria are, therefore, not unique in their ability to express “transgenerational rhythmicity,” although it is likely that the post-translational nature of their core clockwork – which allows for it to keep ticking unimpeded through cell division – makes them a good candidate for transgenerational transmission of temporal information.

In this review, we will discuss the topic of the evolution of circadian clocks, with special focus on clock systems in bacteria for two reasons. First, bacteria evolved before eukaryotes and the selective pressure of daily cycles has existed since the beginning of life on Earth. Therefore, while timekeeping mechanisms in bacteria might have continued to evolve, it is possible that the basic extant machinery is similar to that which first arose (because of the continued selection), and therefore prokaryotic clockworks are likely to be the most similar to an original ancient clockwork that we can access. Second, on the basis of their simpler cellular/genetic organisation, circadian systems in bacteria may be inferred to be the most similar to the first circadian systems that evolved. We will first focus on likely trajectories as clocks continue to evolve, particularly in the context of climate change, and how the spectres of past selection might influence the way that clocks adapt (or fail to do so) in the future. We will then consider the possible adaptive significance of daily timers today, and the approaches that researchers have used to test this adaptiveness. Finally, we will discuss the theoretical background behind our conviction that clocks were adaptive in the past and how the study of bacteria can help us to shine a light upon early clock evolution.

## Spectre of Clock Evolution Yet to Come: Climate Change

*“I am in the presence of the Ghost of Christmas Yet To Come?’ said Scrooge.*

*The Spirit answered not, but pointed onward with its hand.*

*‘You are about to show me shadows of the things that have not happened, but will happen in the time before us,’ Scrooge pursued. ‘Is that so, Spirit?” (Dickens, 1843).*

### Rhythms and Climate Change

The adaptive value of circadian clocks comes from the fact that they allow organisms to appropriately time themselves to consistent daily changes in their environment. By virtue of having a clock, not only can internal events be temporally coordinated within the organism, but the timing of those events can be adjusted to happen at a “local time” of the daily cycle that would be most “optimal” given the environmental rhythm that the organism experiences (e.g., getting the photosynthetic apparatus ready for dawn). In addition to the direct organism-to-environment meshing, the endogenous clock may order events that work better if optimally timed to environmental changes (e.g., oxygen-sensitive nitrogen fixation does not happen at the same time as oxygen-generating photosynthesis, lest the latter would inhibit the former). In essence, clocks allow organisms to anticipate predictable rhythmic changes and sequence biological events to optimally adapt. But what happens when an environment alters characteristics that the biological clock has evolved to anticipate? And what happens if those parameters transmogrify drastically and suddenly, such as in the case of climate change? This is a question some of us might be able to answer within our lifetimes, as climate change provides us with an unwelcome and unprecedented opportunity (Zeebe et al., 2016; Foster et al., 2017).

Human actions have greatly changed our environment, in both physical and biological ways, even before the industrial era. For example, the development of agriculture and animal husbandry – alongside the pre-existing hunter-gathering – during the Neolithic Revolution led to lasting anthropogenic consequences that were already global by 3,000 years ago (Stephens et al., 2019). With agriculture and animal husbandry comes: (i) an increase in human population, (ii) deforestation to make space for crops, livestock and human settlements, and (iii) the spread of invasive species. These human activities had quantifiable effects on the daily rhythms of mammals – which became more nocturnal as their proximity to human settlement increased (Gaynor et al., 2018; for some animals this might have been due to an increase in predation risk, see van der Vinne et al., 2019). Moreover, human selection has also modified the circadian clock of plants, such as the slowing of circadian period and phase delay in cultivated tomato (through mutations in the genes *EID1* and *LNK2*), that likely emerged as an adaptation to the change in its latitude/photoperiod range as humans spread this crop to regions away from its original equatorial home (Müller et al., 2016, 2018). Similarly, a correlation between longer circadian periods and latitudes further from the equator is seen in cultivated soybean, as well as in the wildflower *Mimulus* (Greenham et al., 2017), and comparing *Arabidopsis* accessions from different locations (Michael et al., 2003) has suggested that longer periods are likely selected in higher latitudes. Besides period length, we also see changes in circadian-related genes that regulate flowering time in domesticated crops such as barley (ELF3, Faure et al., 2012) and soybean (PRR3b, Li et al., 2020; GIa, Wang et al., 2016) (for more examples, see McClung, 2021 which reviews this topic). These consequences of domestication indicate that expansion to different latitudes and exposure to new yearly cycles of day length selects for changes in clock genes, particularly in those related to photoperiod-dependent responses. Similar conclusions derive from examinations of circadian and photoperiodic latitudinal clines in a variety of organisms (Kyriacou et al., 2008; Hut et al., 2013 for reviews). These changes are likely to be related not only to the change in photoperiod, but also to the change in the relationship between the yearly cycles of photoperiod and temperature that vary with latitude (see Hut et al., 2013 for this relationship for land and Jabbur et al., 2021 for ocean).

**FIGURE 1:**
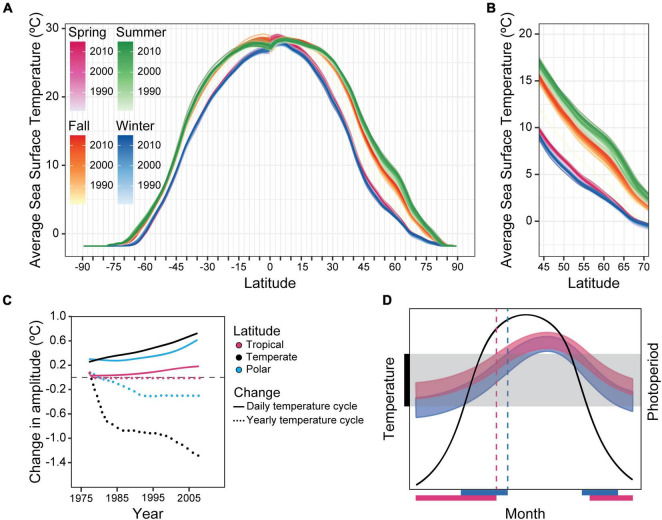
Changes in temperature will likely affect temporal niches. **(A)** Average sea surface temperature (°C) across 34 years (1981–2015) for different latitudes, divided by seasons. Darker colours indicate more recent years. **(B)** Close-up of latitudes 45–70 from the first plot. For these latitudes, the effect of temperature increase seems to be the highest, particularly around summer and fall. In the summer, these temperature increases can be equivalent to a latitudinal shift of almost 5° toward the equator. The temperature data was provided by the NOAA/OAR/ESRL PSL, Boulder, CO, United States, OISST Version 2 (Reynolds et al., 2007), obtained through the International Research Institute/Lamont-Doherty Earth Observatory (IRI/LDEO) Climate Data Library. **(C)** Change in mean temperature and in the amplitude of the daily and yearly temperature cycle for different latitudinal bands. Data modified and redrawn from Wang and Dillon (2014). **(D)** Diagram showing the possible effects of an upwards shift in temperature in the temporal niche of a population. The central bell-shaped curve illustrates the annual change in photoperiod as a function of the month of the year. The black bar on the *y*-axis and the grey shaded area represents the temperature range tolerated by the species. Blue and pink curves represent the yearly temperature cycle for a “baseline” scenario (blue) and a scenario in which the average temperatures increase slightly but the yearly temperature variation decreases (pink), such as in the temperate zone in **(C)**. Blue and pink bars on the abscissa refer to the scenarios explained before and represent the time of the year in which that species is present (e.g., not in summer diapause), based on whether the temperature at the time is within its tolerated temperature range. Dashed lines indicate the photoperiod which anticipates a change from a thermally tolerable to a thermally intolerable condition.

These historical changes might tell us about the selective forces that are currently acting – and will continue to do so – on organisms as a result of post-industrial anthropogenic changes to the environment, as well as climate change; namely, changes in phasing either due to masking or due to changes of major clock properties such as free-running period or the amplitude of the phase response curve (Pittendrigh and Takamura, 1989). A crucial difference between the two situations, however, is the time-scale in which changes are expected to happen: human domestication of the tomato spanned many centuries (Blanca et al., 2012), whereas climate change is modifying the environment in a fraction of that time. Since pre-industrial times (1850–1900), the average global temperature has risen about 1°C, and the latest IPCC report predicts increases in global surface temperature between 1.2–2.0°C and 1.9–3.0°C by 2041–2060, and 1.0–1.8°C and 3.3–5.7°C by 2081–2100 (when compared to temperatures registered between 1850–1900). These predictions are based on the latest scenarios proposed for the smallest vs. the largest projected amount of greenhouse gas emissions, SSP1-1.9 and SSP5-8.5 respectively (SSP = Shared Socioeconomic Pathways; note that SSP1-1.9 includes a transition into negative carbon emissions; IPCC, Masson-Delmotte et al., 2021). If we plot the changes that have already happened in the ocean for different latitudes, we see that, between 1981 and 2015, the average sea surface temperature has increased for all seasons and latitudes (Figure 1A). For some latitudes (Figure 1B), this increase was equivalent to up to a ∼3.5° latitudinal shift toward the equator for the fall, and almost 5° for summer, all within 34 years (in km, this latitudinal shift is equivalent to ∼350–500 km). This by itself is likely to provide a selective force upon circadian and seasonal timing, as now there is a temporal mismatch between important cyclic environmental variables such as temperature and the even more reliably cyclic cue – and previously very reliable predictor of temperature – photoperiod duration. With the predicted increases in temperature as per the Shared Socioeconomic Pathways, the magnitude of this mismatch will increase.

While the average temperatures are increasing, the mismatch caused by this increase is not the only thing that can impose a selective force upon clocks: climate change is also changing the daily and annual *amplitudes* of temperature variation. Between 1975 and 2013, the *daily* temperature variation has increased overall, up to 1.4°C at the poles, 1°C in temperate and 0.3°C in tropical regions, while the *yearly* temperature variation has decreased by 0.6°C (poles), 0.4°C (temperate), and remained unchanged (tropical) (Wang and Dillon, 2014; Figure 1C is based on this study). These observations suggest that while on a daily scale the amplitude of the temperature cycle is increasing, on the annual scale it is flattening. Therefore, not only could the change in temperature due to climate change lead to changes in photoperiod-related clock genes, as was observed for domestication, the data plotted in Figure 1C imply that the selection pressure for circadian clocks might increase with climate change, while that for circannual clocks and perhaps photoperiodism might decrease. These changes in selective pressure through changes in the amplitude of the temperature cycle, however, do not mean that circadian clocks will swiftly become more abundant or conversely that circannual clocks and photoperiodism will no longer be advantageous. Changes in the prevalence of circadian and circannual rhythms, particularly those genetically encoded (rather than mere masking responses), could very well happen at a slow rate. For circannual clocks and photoperiodism, this could be because they might still be beneficial despite a relaxation of the selective pressure toward them. As an example, for species whose reproduction is photoperiodic and restricted to a part of the year, cyclic reproduction might maintain itself simply because it is an evolutionarily stable strategy, especially if the availability of quality food continues to cycle annually. Similarly, given the general complexity of circadian clocks, an increase in selection pressure toward them might still require many generations before clocks with altered properties emerge, or for previously “clockless” organisms (if such there be!) to generate a clock *de novo*.

But changes in the temporal niche, for example, in the times of the year in which an insect is active vs. undergoing diapause – first possibly due to masking and/or plasticity, and later perhaps due to inheritable changes – will likely be very swift; as we can see in Figure 1D, an increase in the average yearly temperature (in this case, associated with a concomitant decrease in yearly amplitude) can shift the time of the year in which a given organism is exposed to temperatures that are within the range it can tolerate (marked by the black bar on the ordinate and grey shading). In the case presented, we observe an overall increase in the temporal niche, represented by the pink and blue bars on the abscissa of Figure 1D (each corresponding to its similarly coloured temperature range) and defined here as the time of the year in which the organism is present or active. We also observe phases of the winter that were previously below the range of tolerated temperatures have now become “available,” or at least thermally tolerable, and the opposite is true for segments of the summer. Finally, the photoperiods that had been useful to predict the transitions between thermally tolerable and intolerable temperatures become “fake news” misinformation (dashed lines), making any genetically hard-wired photoperiodic response misleading as a timer of these ecologically important shifts if it cannot modulate rapidly. This phenomenon would be expected to provide a selective force for organisms to change their sensitivity to photoperiod (e.g., by a shift of its photoperiodic response curve), either through genetics/epigenetics or phenotypic plasticity (Tsai et al., 2020), and therefore climate change could be a major driver toward promoting the evolution of altered clock properties. Note that a change in the preferred season due to climate change may not be as simple as an adaptation to temperature. Many organisms’ life cycles are dependent upon temperature but also upon the availability of food etc., and are delicately choreographed to optimise competing factors. A shift in the temporal niche due to temperature may unbalance an organism from optimal foraging. Thus, climate change could affect organisms on many levels.

### Rhythms, Climate Change, and Bacteria

Some of the complications of trying to predict (and potentially mitigate) the consequences of climate change upon circadian/circannual rhythmicity are that (i) climate change is already underway, (ii) these changes are happening at a pace humans have never previously experienced, (iii) there is not a historical record that can inform and guide us (while paleo-ecology can use the fossil record to estimate the effects of climate change on variables such as ecosystem composition, rhythmicity usually does not “fossilise”), and (iv) laboratory simulations are not realistic for many of the organisms of interest (the long generational time of most of our species of interest means that laboratory experiments simulating climate change would likely be too slow to be of service).

When we think about the effects that climate change will have on the survival of different species, we often think about macroscopic and eukaryotic examples, such as the decline in polar bear populations due to the decrease in sea ice area (Stirling et al., 1999). Or, another example is the increased bleaching of coral reefs – a result of temperatures ≥ 1°C warmer than the usual for the summer – and their observed diminishment in tropical regions concomitant with their “retreat” to the subtropics (Price et al., 2019). And yet another example is the global decline in amphibian populations, due to infection by a fungus that appears to thrive in warmer conditions (Cohen et al., 2019). While those macroscopic examples are indeed very important, climate change also impacts microscopic life such as bacteria, and these in turn are likely to have large-scale effects on other organisms and the ecosystems that depend upon those bacteria, perhaps in heretofore unappreciated ways (Jones and Barbetti, 2012; Evans and Wallenstein, 2014; Gschwendtner et al., 2014; Chase et al., 2021). In this context, the generally rapid life cycles of bacteria could assist climate-change predictions by allowing fast and streamlined laboratory experimental evolution simulations, inspired by the experimental evolution paradigm pioneered by Richard Lenski’s laboratory (Barrick et al., 2009). But how could changes caused by climate change impact the clocks of bacteria?

Interestingly, it is clear that bacteria can be dramatically affected by seasonality. Photoperiod has been shown to be capable of affecting the gut microbial diversity of Siberian hamsters (Bailey et al., 2010; Ren et al., 2020) and sheep (McEwan et al., 2005). In those cases, the effect of photoperiod is indirect – the seasons directly affect the hosts, and the hosts’ seasonal responses affect their microbiomes. In other scenarios, the effect of photoperiodic/seasonal environments directly impinge upon the bacteria. For example, in ice cores, variation in bacterial composition has been associated with monsoon and non-monsoon seasons (Yao et al., 2008). The abundance of marine *Synechococcu*s (a cyanobacterium) shows seasonal patterns in both local and global scales. For an example of a local impact of season, *Synechococcus* levels in a basin in Canada peak around September/October, a little after the highest temperatures are recorded, and decline for about the next 3 months until the lowest temperatures are reached in that environment (Li, 1998). As an example of a global-scale impact of season, the abundance of *Synechococcus* shows a peak during summertime across a wide range of latitudes (Flombaum et al., 2013). In lakes, *Microcystis* (another cyanobacterial genus) has been shown to seasonally produce at least one “anti-freeze” substance that protects them from cold temperatures (Tan et al., 2011). Moreover, the abundance of many lake cyanobacterial species varies with the seasons. If bacteria are also affected by the seasons, it is possible that they experience temporal mismatches caused by global warming such as those projected in Figure 1D.

Following on this point, one particularly significant example is that many lake cyanobacteria have a yearly cycle of abundance, and oftentimes high-density “blooms” are observed during the spring and summer. These blooms are of ecological and economical importance because they can lead to the depletion of oxygen in the lakes and cause the death of the heterotrophic organisms that inhabit them. In addition, there are some species of lake cyanobacteria that produce toxins when they are at high abundance during the blooms, making the water poisonous. A consequence of the deleterious effects of blooms is that their occurrence has been carefully recorded for a long time, and the patterns that these records paint are quite interesting from a seasonal perspective. In Ontario, Canada, for example, there was a significant increase in the number of seasonal cyanobacterial blooms between 1994 and 2009. The cyanobacterial blooms did not just increase in number, however, but also changed their annual timing; they are now occurring later into the summer and autumn than was the case during the 1990s, with some recent episodes happening in late November (Winter et al., 2011). A similar seasonal pattern has been recorded for lakes in the western Alps; analysis of extremely hot weather periods showed that warm autumns and winters promoted cyanobacterial growth, but the same was not true for warm summers. Interestingly, the species composition in those extreme weather events also differed depending on the season (Anneville et al., 2015). Together, these results suggest that the growth (and overgrowth) of cyanobacteria is not only influenced by the increase in temperature, but also by the seasonal timing of that increase, similar to what we proposed in Figure 1D. Given that cyanobacteria are photosynthetic, it is likely that these results are not merely a function of the cyanobacteria’s thermal ranges, but the daylength and intensity of the light in summer vs. winter photoperiods also play a role in these patterns. Finally, there may be seasonal flow of nutrients (e.g., nitrates, phosphates) washed into the lakes by rainfall. Consequently, cyanobacterial blooming is multifactorial, and at least several of these key factors are influenced by climate change and its relationship to photoperiod.

While we have primarily focussed upon the temperature changes that will occur due to climate change, temperature is but one of many environmental variables that have been and will continue to change in the foreseeable future. For cyanobacteria, one of the most important of those is the ocean pH, which has already decreased ∼0.1 from its pre-industrial value of pH 8.2 due to the additional CO_2_ dissolving into the ocean, and is expected to become lower than pH 7.7 by 2100 if current trends continue (SSP5-8.5, IPCC, Masson-Delmotte et al., 2021). And while the acidification of lakes as a result of climate change has been much less studied than that of oceans, lakes have also become more acidic post-industrialisation (Moldan et al., 2013; Weiss et al., 2018). Acidification is very likely to have an impact on cyanobacteria, but accurately predicting the nature of these effects is complicated. While microcosm and mesocosm experiments suggest that acidification should generally favour cyanobacteria in coastal environments (Russell et al., 2013; Ullah et al., 2018), single-species experiments suggest a multiplicity of responses–the growth of some cyanobacterial species is inhibited by acidification while others are stimulated and still other species seem to be impervious to moderate acidifications. Of particular interest is the case of *Trichodesmium*, a nitrogen-fixing marine cyanobacterium that is estimated to be responsible for up to 50% of the oceanic nitrogen-fixation (Mahaffey et al., 2005). Under low pH, the ability of *Trichodesmium* to fix nitrogen is impaired (particularly under the Fe-limited conditions common in the open ocean), possibly because of a reduction in the efficiency of its nitrogenases. This occurs despite the fact that lower pH oceanic water has more CO_2_ dissolved in it, which might be expected to stimulate the growth of cyanobacteria like *Trichodesmium* (Hong et al., 2017). Also inhibited is the carbon/nitrogen fixation of *Nodularia*, *Microcystis aeruginosa*, and *Anabaena spiroides* (the last two species are lake strains). Not all cyanobacteria are inhibited by acidification, however – *Calothrix* is unaffected by acidification, while *Cyanothece*‘s growth and carbon/nitrogen fixation is promoted (Wang et al., 2011; Eichner et al., 2014). The specific effects of the increase in the average pH are thus likely to be very dependent on the particular species and micro-habitat.

Moreover, much like temperature, acidification also varies seasonally with annual modulations of up to 0.08 in pH. These annual changes in pH are highly dependent on latitude, as are the main variables that mediate pH seasonality: temperature in the subtropics and photosynthesis and respiration in high latitudes (Takahashi et al., 2014; Hagens and Middelburg, 2016). For higher latitudes, the pH shows a peak around summertime (becoming more basic), and a trough during winter (becoming more acidic), and the opposite is true for lower latitudes. Similarly to what we described for temperature, however, both latitude ranges are expected to see a decrease in the annual amplitude of their pH rhythm in the future (Kwiatkowski and Orr, 2018). This seasonality, coupled with the overall decrease in oceanic pH and with the pH effects on cyanobacteria growth and nitrogen fixation, could have reverberating effects on the timing/density of cyanobacterial blooms and the seasonal availability of organic nitrogen. These downstream consequences of ocean acidification might exert strong selective forces upon the cyanobacteria, particularly if their timekeepers can also anticipate the rhythm of oceanic pH.

Even if the bacteria themselves are not directly keeping track of the seasons, another important point to consider is that a lot of bacterial species live in symbiotic relationships with organisms that do exhibit anticipatory photoperiodic behaviour, such as the bacteria in mammalian or arthropod gut microbiomes (in some cases, as symbionts). If their hosts’ adaptation is undermined by the temporal mismatch caused by climate change, resident bacteria could also be affected. If this temporal mismatch changes the phase relationship between important variables for the bacteria, such as its host’ feeding rhythm and/or the presence of important hormones like melatonin, the timing of bacterial cycles associated with those variables would likely change and there would be pressure to adapt to the new optimal phase relationship.

A similar phenomenon might happen in free-living bacteria, although they might be more resilient to this type of major change than bacteria that have evolved in tight association with a particular host. This could be the case because free-living bacteria would likely have evolved to rapidly respond and adapt to a wide range of changing conditions in the external environment, while symbionts may be partially insulated by their host from those changes. However, if climate change pushes the bacteria outside of the physiological range they tolerate (e.g., the environment becomes too warm during the day), then even the free-living bacteria could be subject to pressures that are similar to those we suggested for symbionts. On the topic of insect symbionts, many insects - especially those that are food specialists - have mutualistic relationships with bacteria that metabolically aid their hosts (e.g., digest cellulose; Feldhaar and Gross, 2009). For some insects, the presence of the bacteria seems to be very relevant for the hosts’ fitness as the elimination of the bacterial symbionts leads to lower fecundity, shorter longevity, and developmental defects (Douglas, 1998; Baumann, 2005; Zientz et al., 2006). However, the relationship between host and symbionts can be highly dependent on temperature, with high temperatures often causing a decline or demise of the bacterial symbionts. This suggests that these mutualistic relationships could be at risk in a climate-change scenario, thereby altering the fitness of both hosts and symbionts (Wernegreen, 2012; Kikuchi et al., 2016). Similarly, many vertebrates and invertebrates have been shown to have their gut microbiome composition and diversity affected by temperature (Tajima et al., 2007; Chevalier et al., 2015; Berg et al., 2016; Kohl and Yahn, 2016; Bestion et al., 2017; Fontaine et al., 2018; Moghadam et al., 2018; Horváthová et al., 2019; Zhu et al., 2019; Sepulveda and Moeller, 2020). It is unclear, however, whether alterations in the photoperiod or temperature cycles such as depicted in Figure 1D – rather than just a general increase in temperature – could modulate these results.

How will bacterial clocks evolve as the climate changes? What can we learn from them regarding the ways other organisms might respond to climate change, or other long-term changes that modify their environment, leading to a restriction or expansion of their habitat? It is possible that – similarly to coral reefs – they will shift (or expand) their latitudinal range farther away from the equator, particularly if the temperature increase impinges upon their thermal tolerance. *Prochlorococcus* and *Synechococcus*, the two most abundant cyanobacterial genera in the ocean, are expected to increase their abundance in a latitude-dependent way, with both genera expanding to higher latitudes and *Prochlorococcus* in particular experiencing a dramatic increase in the subtropics/temperate zone. Interestingly, *Prochlorococcus* lacks a self-sustained circadian oscillator, presumably due to its tiny size (Chew et al., 2018) and the loss of the gene *kaiA* as a consequence of genome streamlining (Holtzendorff et al., 2008). Could this expansion to higher latitudes, and consequently to a higher variation in yearly photoperiod range, increase selection pressure toward a self-sustained oscillator in *Prochlorococcus*? (Troein et al., 2009). Similarly, as *Synechococcus* increases its abundance in higher latitudes, will they slow down their clock period, as did tomatoes? (Müller et al., 2016, 2018). Or will they take an alternate route and change the sensitivity of their phase response curve to light/dark signals? (Pittendrigh and Takamura, 1989). Will they simply not have enough time to evolve and catch up with climate change, and therefore have to rely instead on phenotypic plasticity to cope with their new environment? How will the “ghosts of their past evolution” to other distinct rhythmic environments constrict the ways they will evolve in the future?

The idea of using bacteria as possible models for understanding climate change is intriguing because studying their evolutionary responses to selective pressures in the lab is a more feasible task than that for macroscopic eukaryotes. In a lab setting, we can expose the bacteria to controlled environments that mimic climate change at a much faster rate and use experimental evolution to determine how they change in those conditions and moreover whether those changes relate to the rate of climate change relative to their generational time. *Escherichia coli* has already been extensively used as a model for this type of experimentation, and through it we have learned that there are many aspects of evolution that can be readily modelled by simply exposing bacteria to different selective conditions and observing the changes that happen to their genome and overall phenotype (Barrick et al., 2009; Good et al., 2017). Interestingly, those experiments observed that evolution is most rapid early in the selective regimen, and within 2,000 generations fitness increases by about 35% (Grant et al., 2020). If these experiments were to be repeated in cyanobacteria such as *Synechococcus elongatus*, we could potentially see similar changes within 6 years under slowly growing conditions (doubling time of about a day), or as little as 3 years under faster growing conditions in which they have been demonstrated to still be capable of circadian rhythmicity (Kondo et al., 1997). Besides the possibility of potentially modelling the impacts of climate change, cyanobacteria can also work as a good proof-of-principle of whether the evolutionary properties that we observe in *E. coli* or other bacteria such as *Pseudomonas aeruginosa* (Huse et al., 2010; Klockgether et al., 2018) can also be observed in cyanobacteria.

Similarly, field studies across different localities and times within the same locality could be made easier by the fact that bacteria’s generally smaller genomes (∼1.4–9.1 Mbps vs. 2.3–149,000 Mbps for eukaryotes) would allow for easier and less expensive metagenomic analyses (Batmalle et al., 2014) for identifying mutations selected by the new forces arising from climate change. One caveat to field studies is the difficulty in determining the relatedness of bacteria collected from different locations in the wild on the basis of morphology or growth characteristics; however, again genomic sequencing may be the answer to assessing how similar/different isolates might be from different regions (e.g., different latitudes). Bacteria have been fundamental in helping us dissect how organisms use their circadian clocks to anticipate predictable conditions, thus it will be interesting to discover what they can teach us about how plastic clocks may be as they evolve in response to new selective challenges.

## Spectre of Clock Evolution Present: The Current Adaptive Value of Circadian Clocks

*“Come in, – come in! and know me better, man! I am the Ghost of Christmas Present.*

*Look upon me! You have never seen the like of me before!” (Dickens, 1843)*

Having considered how clocks might cope with a temporally mismatched future, let us now shift our focus to the present, and discuss the issue of the current adaptive value of circadian clocks. As mentioned before, clocks are believed to be adaptive because they can allow organisms to anticipate consistently occurring environmental conditions and possibly phase important aspects of their behaviour, metabolism, or physiology to the “most optimal” time of the day or the year. The importance of this phasing can be inferred from models and from actual competition experiments, for example between strains of cyanobacteria with different circadian periods, in which the out-competing strain is always the one whose endogenous period resonates optimally with the period of the environmental cycle (Ouyang et al., 1998; Hellweger, 2010; Ma et al., 2013). Similar results were observed for plants (Dodd et al., 2005) and mice (Spoelstra et al., 2016), and partially in fruit flies (Horn et al., 2019).

This is, however, not the only way that clocks could increase the fitness of their owners. Another way that clocks can be adaptive is by establishing an internal temporal order, that is, making it so that internal events such as cell division, photosynthesis or nitrogen fixation happen in a synergistic series. Quoting the late chronobiologist Serge Daan (Daan, 1987), “an animal performing all its activities in optimal proportions but in random temporal sequence would be continuously taking the right decisions at the wrong time.” The timing of periodic behaviours in relationship to the environment matters, but so does the order of internal events. Thus, we are left with two main hypotheses as to how clocks might be able to increase fitness and have therefore evolved and been maintained as an adaptation, namely that (i) clocks allow for optimal timing with the environment; and (ii) that even in the absence of a cyclic environment, clocks orchestrate a harmonic internal temporal order. Both of these benefits could lead to increases in reproductive success and overall fitness, and they are not mutually exclusive; both could be simultaneously operating in the same organism at the same time. But how can we test if either or both of them actually do so?

Testing whether clocks are adaptive and defining the mechanism underlying that adaptiveness requires first and foremost a rigorous assessment of whether clocks increase fitness. Prior to the pioneering “competition” studies in cyanobacteria, it was not apparent how to accomplish that task. While one could demonstrate, for example, that having a clock which resonates with the environment – or even having a clock at all – could increase growth (Ketellapper, 1960), survival, or lifespan (Pittendrigh and Minis, 1972; von Saint Paul and Aschoff, 1978; Hurd and Ralph, 1998; Klarsfeld and Rouyer, 1998), fitness is unfortunately not always directly correlated with any of those variables (Endler, 1986). For example, an organism that survives for a long time but fails to reproduce would have a fitness of zero. Conversely, another organism that lives for a short amount of time could be assumed to have a low fitness based on its lifespan alone, but if its successful reproductive rate was high, then its fitness would be larger than if it lived for a long time but reproduced inefficiently. Then again, a high number of offspring is not solely indicative of high fitness, for a high reproductive rate coupled with very little parental investment might well lead to fewer offspring who successfully reproduce in the next generation than one would initially assume based on number of offspring alone. It is complicated! In summary, in the absence of an experimental organism in which we can observe the frequency of a given phenotype in a population throughout multiple generations, demonstrating the adaptive significance of clocks is a tricky endeavour.

**FIGURE 2:**
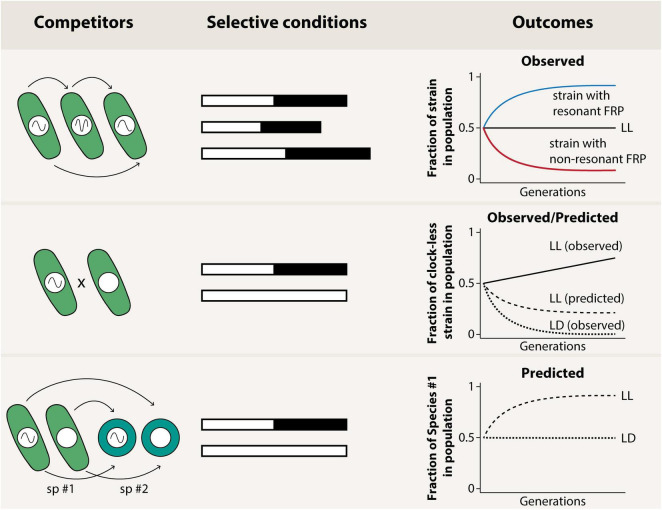
Diagram showing the results of experiments done previously to test the adaptive value of the circadian clock in cyanobacteria **(first** and **second row)**, as well as a proposed experiment **(third row)**. Experiments in the first row refer to publications described in the text (Ouyang et al., 1998; Woelfle et al., 2004; Ma et al., 2013), and show that resonance between the circadian period (FRP = free-running period) and the environmental period increases cyanobacterial fitness, while no difference was observed in constant conditions (LL). Experiments in the second row are explained in more detail in the text (Woelfle et al., 2004; Ma et al., 2013), and show that cyanobacteria with circadian clocks outcompete clock-less cyanobacteria in rhythmic (LD) but not in constant (LL) conditions. This latter result was unexpected given the typical bias among chronobiologists that circadian clocks would be able to increase fitness by facilitating proper internal temporal order. The last row features an experiment that is yet to be reported, namely, interspecific competitions between different species of cyanobacteria. A possible prediction of this experiment is that the co-existence or not of the two species would be modulated by the environmental rhythmic conditions, and perhaps by the two species’ circadian clocks.

The establishment of cyanobacteria as a clock model introduced an experimental preparation to which a population biologist’s “competition” test of adaptive fitness could be applied relatively easily. With cyanobacteria in a homogenous cell suspension, direct measurements of fitness become feasible, and this pioneering paradigm has now been extended to plants and animals. This test demonstrated the existence of an optimally adaptive relationship between the period of the circadian clock and the period of the environmental cycle (in nature, 24 h) (Figure 2, first row; Ouyang et al., 1998). This optimal relationship is presumably based on the entrainment characteristics of the circadian clock to the environment to synergise an adaptive phasing of the cell’s temporal programme. Similarly, having a self-sustained clock in a cyclic environment was shown to be adaptive, as arhythmic strains or strains with a damped oscillator were out-competed by wild-type in a 24 h light/dark cycle (LD 12:12). These results gave support to the first hypothesis mentioned, that is, that clocks are adaptive (Woelfle et al., 2004; Ma et al., 2013; Welkie et al., 2018) and that their adaptiveness comes from the fact that they allow optimal phasing (Ouyang et al., 1998; see Lambert et al., 2016 for a possible explanation of why phasing is important). Support for the second hypothesis, however, was not found in the cyanobacterial experiments: in constant conditions – in which optimal phasing is no longer relevant because there are no environmental changes to “phase to” – arhythmic cyanobacteria strains slowly outcompete those with clocks, suggesting that the internal temporal order present in wild-type might not increase its fitness, at least not in relationship to the particular arhythmic mutant tested and in “comfortable,” low-stress laboratory conditions (Figure 2, second row).

Do these results mean that internal temporal order is irrelevant in cyanobacteria? Perhaps. But this conclusion has one important caveat: while the cyanobacteria experiments that demonstrate that the *phasing* of the clock is adaptive have found parallels in other organisms (Pittendrigh and Minis, 1972; Hurd and Ralph, 1998; Klarsfeld and Rouyer, 1998; Dodd et al., 2005; Spoelstra et al., 2016), most of the non-cyanobacterial work that examined the adaptive value of the clock did so in cyclic conditions only, and thus we cannot evaluate whether the result in which an arhythmic cyanobacterial strain outcompeted wild-type in constant conditions is reproducible across multiple taxa. One of the few studies that competed both wild-type and arhythmic mutants in constant conditions was done in the fruit fly *Drosophila melanogaster*, and it was observed that arhythmic per^0^ mutants lose against wild-type both in cyclic and non-cyclic conditions, in stark contrast to the reported cyanobacterial result (Woelfle et al., 2004; Horn et al., 2019).

Which of these data represent the most phylogenetically common response? It is hard to say. Some of the *Drosophila* results were unexpected, as flies with a long-period were able to outcompete wild-type flies in a long T-cycle, but the same was not true for flies with a short-period in short T-cycles, a result that is only partially consistent with previous reports. At the same time, it could be argued that since cyanobacteria are photosynthesising organisms, perhaps a loss of fitness due to poor internal temporal order can be compensated by an increase of fitness because arhythmic cells that have a direct non-anticipatory response to light/dark are not as disadvantaged as those cells that have an innate “off phase” for their photosynthesis. Observations of the rhythm of photosynthetically produced oxygen in LD and LL suggest that that might not be the case, however: while some cyanobacterial species like *Synechocystis* PCC 6803 and *Cyanothece* RF-1 appear to show rhythmic photosynthesis in LL, photosynthetic activity in LL is essentially constitutive for *Synechococcus* PCC 7942 – the strain used in the competition experiments – at least as measured by the levels of photosynthetic oxygen evolution (Yen et al., 2004). Either way, with so few examples, it is currently impossible to draw strong conclusions about the general adaptiveness of the circadian clock choreographing an “internal temporal order.” However, in more recent experiments using a *kaiABC* deletion strain – rather than the CLAb point mutation of *kaiC* (which was the “clock-less” strain tested in the competition experiments mentioned before, Woelfle et al., 2004) – we have observed that wild-type strains outcompete the clock-less *kaiABC* deletion strains in LL (our unpublished data), in line with the *Drosophila* results (Horn et al., 2019). This incongruency in results might be a consequence of the fact that the two different “clock-less” strains used in these experiments achieve phenotypic arhythmicity through different mechanisms, and that a point mutation of *kaiC* (but not complete clock deletion) could lead not only to arhythmicity but also to previously unconsidered phenotypes that aid survival and/or reproduction.

As research into bacterial chronobiology expands, however, we might soon be able to use non-photosynthetic bacteria to further address this question – in the last 5 years, two other bacterial species have emerged as possible clock models. One is the gut bacterium *Klebsiella aerogenes* (Paulose et al., 2016, 2019), [curiously, another *Klebsiella* had been reported to have rhythms in 1973 (Sturtevant, 1973), but the report failed to demonstrate defining criteria of circadian clocks]. The second recent example is the soil/gut bacterium *Bacillus subtilis* (Eelderink-Chen et al., 2021). These two bacteria could allow for a similar competition test of adaptive significance as has been performed in cyanobacteria, but without the complication incumbent in using cyanobacteria for which their entraining factor is also their main source of energy, i.e., light! As neither *Klebsiella aerogenes* nor *Bacillus subtilis* harbour a KaiC-based oscillator (*Bacillus* lacks *kaiC* totally, and while *Klebsiella* has genes that are similar to *kaiABC*, they are not genetically orthologous, Paulose et al., 2016), they will also be interesting for tracing different evolutionary paths and/or pressures that could lead to the evolution of circadian clocks. In particular, the presence/absence of light is obviously very important for multiple functions in photosynthetic cyanobacteria, but what is the daily selective pressure(s) for heterotrophs like *Klebsiella aerogenes* and *Bacillus subtilis*? Similarly, studies involving other cyanobacteria can further elucidate the evolution and adaptive significance of the clock, particularly if we employ not only the work-horse cyanobacterium *Synechococcus elongatus* PCC 7942, but other cyanobacterial species as well. Cyanobacteria that also express circadian rhythms such as *Synechocystis* PCC 6803 and *Anabaena* PCC 7120, and that grow under the same conditions can help to confirm our previous results. If different cyanobacterial species are competed amongst each other, the results may reveal the *inter-*specific adaptive value of the clock (Figure 2, third row), as well as how the circadian clock and the environmental cycles modulate inter-specific interactions. Species with an incomplete *kaiABC* clock cluster like the genomically streamlined cyanobacterium *Prochlorococcus*, which had a secondary loss of KaiA (retaining only a non-functional truncated form of KaiA) (Holtzendorff et al., 2008), or *Gloeobacter violaceus* and the recently described *Anthocerotibacter panamensis* (Rahmatpour et al., 2021) – which are cyanobacteria that lack all *kaiABC* genes – can also help to establish the specific conditions in which a completely self-sustained clock has a higher fitness than a damped oscillator or a naturally clock-less system. Moreover, studying these other cyanobacterial species could point toward possible ways that selection favoured circadian clocks and made them so phylogenetically widespread.

## Spectre of Clock Evolution Past: The Primaeval Circadian Clock

*“I am the Ghost of Christmas Past.’*

*‘Long Past?’ inquired Scrooge.*

*‘Your past,’ replied the Ghost.” (Dickens, 1843)*

What were the early Earth conditions that led to the emergence of circadian clocks? This question has a “lot of moving parts.” To attempt an answer, we must first consider the existing evidence for multiple origins of circadian clocks; while clocks themselves are widespread in the tree of life, the genetic bases underlying them are wildly variant, and little homology is found as one moves across taxonomical scales (although Edgar et al., 2012 suggested a broadly conserved, peroxiredoxin circadian clock machinery – a hypothesis that is not widely accepted at the present time). Granted, there is general homology within Metazoa of clock genes such as *Period*, *Cryptochrome*, *Timeless*, *Bmal1/Cycle*, *Clock/Jerk* etc. (Tei et al., 1997; Rutila et al., 1998). Homologues of key genes such as *Bmal1*/*Cycle* have not been found in sponges (Müller et al., 2013), although they are present in cnidarians (Reitzel et al., 2010; Hoadley et al., 2011), suggesting that the Metazoan clock as we commonly know it might have evolved sometime around the divergence between Porifera and Cnidaria. Just below Metazoa on the “Tree of Life,” we land upon Ophistokonta and a new set of functionally diverse oscillator genes in fungi, namely the rather conserved WC-1 and WC-2 (white collar), FWD-1 (F-box/WD-40 domain containing protein-1), FRH (FRQ-interacting RNA helicase), and the less conserved but well studied FRQ (frequency) genes (Crosthwaite et al., 1997; Salichos and Rokas, 2010). None of the fungal clock genes mentioned are obvious homologues of Metazoan circadian genes. Even further down the trunk of the Tree of Life, we enter the domain of plants and their CCA1-TOC1 based oscillators, which are highly conserved among plants and present from angiosperms to green algae such as *Ostreococcus tauri* and the charophyte *Klebsormidium flaccidum* (Linde et al., 2017), suggesting broad conservation among Viridiplantae. Those genes, however, are not homologues of any of the major clock genes in Metazoa or fungi that were described above. The single gene product that appears to be involved to some extent in the clocks of all eukaryotes is the kinase *Casein Kinase* Iε/δ and its homologues (Fan et al., 2009). Finally, while the genetic basis of the clocks of basal eukaryotes (e.g., Excavata) has yet to be properly identified, it seems unlikely that once resolved, we will encounter homology between their clock genes and those of other Eukarya. Additionally, while the KaiABC-based oscillator seems to be almost ubiquituous in cyanobacteria and the *kaiC* and *kaiB* genes appear to be widely distributed among Eubacteria and present in some Archaea, the other two bacteria in which circadian rhythms have been reported (*Klebsiella aerogenes* and *Bacillus subtilis*) are two Eubacteria that lack *kai* genes. As of this writing, *kaiABC* homologues have not been observed in any eukaryotes (not even in chloroplast genomes). The bottom line is that the genetic evidence suggests multiple independent origins of circadian machinery during evolution with convergence upon very similar functional properties.

Therefore, assuming that cyanobacteria, plants, fungi, and Metazoa all emerged at very different geological times, and that the environmental conditions on earth were dramatically different at those times (especially between cyanobacteria’s first emergence as compared with that of higher eukaryotes), answering the question of what conditions constitute a selective pressure(s) that could have encouraged the emergence of circadian clocks requires answering multiple versions of that same question. Here, we will only attempt to answer one of those versions: specifically, what were the early Earth conditions that could have acted as selective pressure(s) for the emergence of circadian clocks in cyanobacteria? One of the main ideas regarding circadian clock evolution posits the “Escape from Light Hypothesis,” which postulates that the clock evolved as a response to the predictable daily change in light radiance (Pittendrigh, 1993). Given the fact that ultraviolet radiation (aka UV) and other wavelengths of light can induce DNA damage and eventually lead to the accumulation of mutations, one could expect that there would be selection to restrict the timing of DNA replication – which requires chromosome unwinding and thereby may make the DNA molecule more exposed to UV damage – to the part of the day in which UV radiance is the lowest (Pittendrigh, 1993; Nikaido and Johnson, 2000).

**FIGURE 3:**
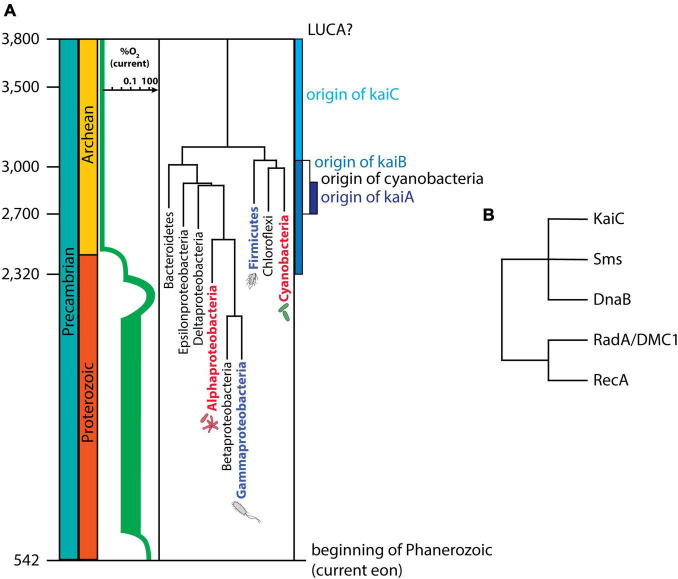
Evolution of the cyanobacterial circadian clock genes. **(A)** Timeline of the evolution of the three core cyanobacterial clock genes, *kaiA*, *kaiB*, and *kaiC*, compared with the timing of the Great Oxidation Event [in green, oxygen levels in relationship to today’s atmosphere (based on Lyons et al., 2014)], and the evolution of Eubacteria (timing based on estimates by TimeTree (Hedges et al., 2015; Marin et al., 2016) added for evolutionary context. The date of the origin of *kaiA* is directly extracted from recent studies (Dvornyk and Mei, 2021), while dates for *kaiB* and *kaiC* are based on previous work (Dvornyk et al., 2003) after adjusting for the most current estimates of the origin of Cyanobacteria. All taxons represented in the figure contain copies of *kaiC*, although not all groups with *kaiC* are shown, and non-*kaiC*-containing groups are not included for simplicity. Labels shown in red are bacterial groups that have a KaiBC-based timer [a KaiABC-based circadian clock in the case of Cyanobacteria, and a KaiBC “proto-clock” (hourglass timer?) in the case of *Rhodopseudomonas* (Ma et al., 2013)]. Blue-font labels indicate other Eubacteria in which a potential circadian clock has been described: *Bacillus subtillis* (Firmicutes) and *Klebsiella aerogenes* (Gammaproteobacteria). The origin of cyanobacteria is based on estimates by Blank and Sanchez-Baracaldo (2010), which is in general agreement with other reports. LUCA refers to the Last Unique Common Ancestor. **(B)** Protein tree of KaiC and other proteins in the RecA/DnaB superfamily (based on Leipe et al., 2000; Makarova and Koonin, 2017).

Chronologically speaking (Figure 3A), the origin of cyanobacteria is estimated to have occurred around ∼3,000 Mya. Because all *kaiC* genes are monophyletic, the emergence of *kaiC* would have taken place between the last common ancestor of Archaea and Eubacteria (aka LUCA, for the “Last Unique Common Ancestor”), and the origin of Cyanobacteria (currently, the data support the hypothesis of *kaiC* origin in Eubacteria and then lateral transfer to Archaea, see Dvornyk et al., 2003). Based on the dating of these events (Blank and Sanchez-Baracaldo, 2010), we can estimate that the circadian clock gene *kaiC* first emerged some time between 3,800 and 3,000 Mya (Figure 3A). This estimate is an update from previous work (Dvornyk et al., 2003) that estimated the origin of *kaiC* between 3,800 and 3,500 Mya, but took the origin of cyanobacteria to be 3,500 Mya, an estimate that was derived from now-controversial cyanobacterial microfossil evidence (Schopf and Packer, 1987; Brasier et al., 2002; Wacey et al., 2016). The original *kaiC* likely derived from the RecA/DnaB superfamily (Figure 3B, Leipe et al., 2000), which is highly involved in DNA repair and possibly was already present in LUCA (Chintapalli et al., 2013). This origin is in line with the Escape from Light hypothesis, as it suggests that KaiC might have initially been associated with DNA replication, DNA damage repair, and/or RNA metabolism (for example, KaiC shares some structural/sequence similarities with RNA helicases). Interestingly, when compared to other proteins in that family (Figure 3B), KaiC is unique in that it does not possess obvious DNA-binding motifs, and it does not seem to be capable of binding to DNA. While one report (Mori et al., 2002) suggested that KaiC might be capable of binding forked DNA, this observation has not been followed up, and there is scant additional evidence that KaiC is capable of binding directly to DNA. Why exactly KaiC would lack a DNA binding motif is uncertain, but perhaps DNA binding through intermediate linkage kinases (SasA and CikA) and a transcriptional factor (RpaA) allows more flexibility and additional regulatory options than does a direct DNA-binding mechanism.

The presence of KaiC alone, however, is not sufficient to generate a circadian timer in cyanobacteria, but perhaps it could have generated something akin to an hourglass when it first diverged in the RecA/DnaB superfamily (Johnson et al., 2017). Around this geological time as well (∼3,000 Mya), UV radiation reaching the surface of the Earth was much stronger than it is nowadays. While the Sun was ∼25% less luminous, our star likely emitted ∼4 times more UV radiation, and Earth still did not have an ozone layer that could attenuate that radiation. Estimates from the predicted atmosphere from the early Archean Eon (3,500 Mya) and late Archean Eon (2,700 Mya) suggest that UV irradiances were 2–3 orders of magnitude higher than today’s levels in the 200–280 nm (UV-C) range. Considering the action spectrum for DNA damage (with a peak in the UV-C; Green and Miller, 1975), and even though the duration of a day was shorter at that time (considered here as ∼14 h, although this value is highly contentious, see Spalding and Fischer, 2019), the daily fluence of UV-C would be about ∼500 times greater per day than the current levels (see Cockell, 2001, for a full explanation). This calculation assumes cloudless skies and an atmosphere free of any UV absorbers. And while this estimate is likely to be more of an upper limit than the average UV daily fluence, it does suggest that UV damage to DNA would have been very intense during those primordial eras, and there would likely have been strong selection for any UV-sensitive process (such as replication?) to be regulated so as to occur exclusively at night. Likewise, even when we consider possible UV absorbers in the seawater such as Fe(III)-silica precipitates, UV radiation was still high enough to greatly impair cyanobacteria’s growth and expansion in those environments (Mloszewska et al., 2018). A KaiC-based hourglass or a KaiBC damped oscillator that temporally restricted light/UV-sensitive events to the nighttime in accordance with the “Escape from Light Hypothesis” would likely provide a fitness benefit under such conditions.

Initially, it was thought that *kaiA* evolved considerably after *kaiC* and *kaiB*, around ∼1,000 Mya (Dvornyk et al., 2003). This was the interpretation because: (i) *kaiA* is exclusive to cyanobacteria and (ii) even some cyanobacteria lack it. Recently, with more available genomes to analyse, many of the cases of “*kaiA* absence” were found to be secondary losses, and the origin of *kaiA* was pushed back to around 2,900–2,600 Mya (Dvornyk and Mei, 2021), closer to the origin of cyanobacteria at around 3,000 Mya (Blank and Sanchez-Baracaldo, 2010). This would place it shortly before the Great Oxidation Event (GOE), which is thought to have begun between 2,400 and 2,050 Mya. During the GOE, the atmospheric oxygen levels rose sharply – an increase attributed to photosynthetic oxygen production by cyanobacteria – turning the weakly reducing atmosphere into a strongly oxidising environment. This likely caused a major extinction event, as oxygen is toxic to species that can neither combat reactive oxygen species (ROS) nor elude the oxygen by migration to a low-oxygen region. Moreover, the GOE enabled the later formation of the ozone layer in the atmosphere around 600 Mya, which began to absorb a considerable amount of UV radiation. This timeline implies that, throughout the evolution of all three cyanobacterial clock genes, intense UV radiance was likely to be present, and therefore a strong candidate for a selective force.

Within this new timeline, *kaiA* would also have evolved prior to the origin of multicellularity in cyanobacteria (e.g., filamentous multicellular cyanobacteria), and certainly before cyanobacteria reached their maximum morphological complexity (Blank and Sanchez-Baracaldo, 2010; Schirrmeister et al., 2011). While the cyanobacterial literature covered in this review has thus far focused on unicellular model cyanobacteria such as *Synechococcus* and *Synechocystis*, filamentous cyanobacteria have also been shown to have daily (Stal and Krumbein, 1985) and circadian (Chen et al., 1998) rhythms. Given the fact that a circadian clock allows not only for intra-cellular temporal coordination, but also for inter-cellular temporal coherence between cyanobacterial cells in a filament (Arbel-Goren et al., 2021), it is tempting to think that the circadian clock facilitated the cell-to-cell coordination that would be required for multicellularity. It could also be argued that the presence of a clock would make it possible for bacteria to better temporally partition their niche, eventually increasing co-existence and diversity. But while these speculations suggest ways that KaiA might have influenced the evolution of cyanobacteria, they do not address the question of which selective forces might have led to the emergence of *kaiA*.

What we know about the current KaiABC oscillator implies that the emergence of *kaiA* allowed cyanobacteria to move from a damped KaiBC oscillator to a self-sustained oscillator, potentially allowing for more flexibility in phasing (Johnson et al., 2017; Kawamoto et al., 2020). Why would this greater flexibility be important? Previous work has implicated the role of the yearly variation in day length as a selective factor for self-sustained oscillators (Troein et al., 2009; Hut and Beersma, 2011; Johnson et al., 2017). Unfortunately, we have no way of knowing the biogeography of early Earth cyanobacteria, but it is possible that expansion to different latitudes would have selected for self-sustainability and led to the emergence of *kaiA*. The current global distributions of *Synechococcus* and *Prochlorococcus* (Flombaum et al., 2013) tempt us to conclude that the wider latitudinal distribution of *Synechococcus* was enabled by its full-blown self-sustained KaiABC oscillator which thereby made them able to adapt optimally to higher variation in yearly photoperiod. On the other hand, *Prochlorococcus* is an abundant cyanobacterium that has not colonised the highest latitudes in the oceans, possibly because it is restricted by the lack of functional *kaiA* and consequently has a damped oscillator phenotype (Holtzendorff et al., 2008; Kawamoto et al., 2020). Likewise, a corroborative example comes from the recent description of a very ancient member of Gloeobacteria (sister group to Crown Cyanobacteria), *Anthocerotibacter panamensis*, that was isolated from equatorial regions (Rahmatpour et al., 2021). *A. panamensis* lacks all the *kai* clock genes found in other cyanobacteria and this correlation with its equatorial distribution suggests that an expanding biogeography might have selected for the evolution of the KaiABC oscillator. Yet, all of these observations do not imply obligatory causation since *Anthocerotibacter*’s sister group *Gloeobacter*, which also lacks *kaiABC*, is capable of living in temperate zones (Rippka et al., 1974). More will be revealed by further research.

Could the spectral remnants of clock evolution still be detected in extant cyanobacteria? The cyanobacteria that live on Earth today are not the same as the cyanobacteria that lived 3,000 Mya. Perhaps that is one of the complications of using cyanobacteria to answer questions of evolution: while they do give us insights into what was likely a primaeval clock mechanism, so much evolutionary time has passed that many of the traces of this evolution are likely to have been obscured by more recent invention in response to changing environments and new competitors. But perhaps we can make use of experimental evolution experiments to identify what might have been the evolutionary pathways and effective selective pressures that can lead to a circadian clock – and perhaps even seasonal timing. Cyanobacteria or another bacterium might be the most suitable candidate model organism for this type of endeavour.

## Summary and Concluding Remarks

In this review, we have discussed the past, present and future of the evolution of circadian clocks. Phylogenetically widespread, these clocks show very little genetic conservation across major taxa, suggesting multiple evolutionary origins, convergent evolution, and high diversity. While we cannot say how and why each circadian clock evolved, we gain perspective on what might have been the selective forces that guided their evolution through the study of cyanobacteria. These ancient prokaryotic organisms possess three clock genes, *kaiA*, *kaiB*, and *kaiC*, which evolved very early in the Tree of Life, at a time when there was little protection against UV radiation, and thus selection toward DNA protection from UV damage was likely to be very strong. One way cells can defend against UV damage to their DNA is by restricting the timing of their DNA replication to the night, as posited by the Escape from Light Hypothesis. KaiC’s relatedness to RecA and DnaB proteins suggests that indeed KaiC could have materialised from a DNA repair background. KaiC alone, however, is not enough to generate a self-sustained oscillation, and the emergence of *kaiB* and *kaiA* were crucial to creating the circadian clockwork as we know it today. We hypothesise that the yearly variation in photoperiod could have been the evolutionary pressure that made self-sustained circadian oscillators more advantageous than proto-clock timers based on just KaiC or KaiBC. Unfortunately, as we lack a fossil record that tells us about the biogeography of cyanobacteria, we only have correlations to support this hypothesis. Nevertheless, given cyanobacteria’s fast generation time and ease of cultivation, this hypothesis could be tested through experimental evolution experiments in which cyanobacteria that naturally lack *kaiA* or *kaiA* and *kaiB* (or cyanobacteria in which these genes have been knocked-out) are grown for multiple generations under either static or varying photoperiods. If indeed self-sustainability is more advantageous when photoperiods vary, we would predict the emergence of mutations that lead to a self-sustained phenotype in the cells exposed to varying, but not static, photoperiods. Whether this hypothesis is true or not, testing it could provide us with useful insights on circadian clock evolution, as well as guide our efforts in the search for other prokaryotes that might harbour *bona fide* circadian clocks.

We also discuss herein the current adaptive value of circadian clocks as assessed by competition experiments, and summarise research done initially in cyanobacteria, and later extended to higher eukaryotes like *Arabidopsis*, *Drosophila*, and mice. Generally speaking, it is thought that circadian clocks can increase fitness primarily through: (i) allowing anticipation of important cyclic environmental conditions, thus permitting optimal timing of activities such as foraging or photosynthesis, and (ii) establishing an optimal internal temporal order. We find taxonomically broad support for the first proposition (Ouyang et al., 1998; Dodd et al., 2005; Hellweger, 2010; Ma et al., 2013; Spoelstra et al., 2016), but tests of the second proposition have been restricted to cyanobacteria and *Drosophila*, with currently conflicting results (Woelfle et al., 2004; Horn et al., 2019). However, testing for the adaptive value of an internal temporal order is conceptually simple, and would generally require only that the experiments previously discussed be repeated under constant conditions.

Finally, while many questions about the past and the present evolution of circadian clocks still remain to be answered, we currently face a new spectre: climate change, which will mould and direct the future of clock evolution. As the environment changes at an unprecedented rate, many of the environmental cycles that are important to an organism’s survival and to which circadian clocks sync will rapidly transmute. We particularly note the changes in the amplitude of the daily and yearly temperature cycles (Figure 1C), as well as changes in the relationship between the annual variation in photoperiod and temperature (Figure 1D). But other changes are also likely, particularly as further deforestation, coupled with the increase in global temperatures, leads to desertification, droughts and more extreme climatic events. All of this greatly undermines the previously reliable cue of day length, initially making it a source of “misinformation” about the future and later possibly leading to a shift in how organisms respond to particular photoperiods, as depicted in Figure 1. We are already seeing the effects of these changes upon rhythmic events, such as bird migration or algal blooms. However, throughout the rest of the current century, we will very likely continue to experience further temperature increases that will lead to increasingly severe temporal mismatches between cues like photoperiod and important variables such as temperature. How fast and how much will clocks be able to adjust their properties? What will be the impact on the rest of the ecosystem if they do adapt, or if they fail to do so? It is imperative that we use the knowledge currently available about clock evolution, as well as the experimental and modelling tools we have at hand, to determine how clocks will and will not change, and how these changes will affect the biotic and abiotic components of their ecosystem. As Ebenezer Scrooge learned in *Christmas Carol*, if the lessons of spectres are taken to heart, the phantasmagoria revealed by the Future may be averted. Perhaps we can learn from the Spectres of Past and Present and change the fate of the shadows that are upon us. Perhaps.

## Author Contributions

MLJ and CHJ wrote the manuscript. Both authors contributed to the article and approved the submitted version.

## Conflict of Interest

The authors declare that the research was conducted in the absence of any commercial or financial relationships that could be construed as a potential conflict of interest.

## Publisher’s Note

All claims expressed in this article are solely those of the authors and do not necessarily represent those of their affiliated organizations, or those of the publisher, the editors and the reviewers. Any product that may be evaluated in this article, or claim that may be made by its manufacturer, is not guaranteed or endorsed by the publisher.
